# Supplementary material for: The Distribution Characteristics of Aerosol Bacteria in Different Types of Pig Houses
Source: Animals (Basel). 2022 Jun 14;12(12):1540. doi: 10.3390/ani12121540 (PMC9219456; doi:10.3390/ani12121540)
Supplement: Supplementary file 1 [file animals-12-01540-s001.zip › animals-1733972-supplementary.pdf]

Supplementary Table S1. Relative abundances of the airborne bacterial genera (%).

Bacterial genera	FAR	WEA	FAT
Clostridiales	21.70	5.48	29.44
Psychrobacter	16.07	0.08	1.95
Streptococcus	6.78	12.78	1.74
Terrisporobacter	4.96	1.60	3.40
Lactobacillus	4.47	16.24	2.12
Corynebacteriaceae	2.71	4.14	9.97
Aerococcus	2.03	1.70	5.49
Cyanobacteria	1.88	2.54	1.87
Staphylococcus	0.58	0.72	9.56
Prevotellaceae	0.06	4.73	0.12
Others	38.76	49.99	34.35
